# EDITOR’S MESSAGE / MESSAGE DE LA RÉDACTION

**DOI:** 10.29173/jchla29819

**Published:** 2024-12-01

**Authors:** Jessica McEwan

**Affiliations:** JCHLA/JABSC Editor

*[Français en suite]*

As the incoming Editor of the JCHLA/JABSC, I would like to recognize and thank former Editor, Megan Kennedy, for her work on the editorial board over the last three years.

In the months ahead, I look forward to working with the 2024-2025 editorial board whose time and expertise contribute to making this publication possible: Ashley Farrell as Senior Editor, Melissa Walter as Junior Editor, Tyler Ostapyk as Production Editor, and Katherine Merucci as Copy Editor.

This issue opens with a column penned by CHLA/ABSC President, Amanda Ross-White, and Jeff Mason titled *Canadian health library cuts and closures: what CHLA/ABSC is doing, and what you can do too*. It explores recent advocacy work CHLA/ABSC has engaged in on behalf of its members and shares approaches to advocacy that those of us in health information roles can put into practice too.

We have three research articles in this issue:

- Alexander, Hall, and Chen explore librarian involvement in knowledge synthesis articles and its relationship to citation counts and the Journal Impact Factor.
- McKeown, Hopman, and Roy compare in-person classroom instruction to online video instruction for teaching literature searching skills to graduate students.
- Visintini and McEwan showcase lightweight, user experience space assessment strategies in a small hospital library context to support furniture upgrades and library policy.

In book reviews, Leonard considers Crum and Nunez’s 2023 *Essential leadership skills for health sciences information professionals* while Nault reviews Jamison’s 2024 *Decentering whiteness in libraries: a framework for inclusive collection management practices*.

Our issue closes with a product review by Hilton of the Joanna Briggs Institute’s *Critical appraisal checklist for systematic reviews and research syntheses* that examines its utility in evaluating the research methodologies of systematic reviews.

I hope you enjoy this issue of the JCHLA/JABSC!

**Jessica McEwan**

*JCHLA/JABSC Editor*

*Email:* editor@chla-absc.ca

En tant que nouvelle directrice de la rédaction JABSC/JCHLA, j'aimerais remercier la directrice de la rédaction précédente, Megan Kennedy, pour son travail au sein du comité de rédaction au cours des trois dernières années.

Dans les mois à venir, je me réjouis de travailler avec le comité de rédaction 2024-2025, dont le temps et l'expertise contribuent à rendre cette publication possible : Ashley Farrell, rédactrice principale, Melissa Walter, rédactrice adjointe, Tyler Ostapyk, rédacteur de la production, et Katherine Merucci, rédactrice réviseuse.

Ce numéro s'ouvre sur une contribution rédigée par la présidente de l'ABSC/CHLA, Amanda Ross-White, et Jeff Mason, intitulé *Canadian health library cuts and closures: what CHLA/ABSC is doing, and what you can do too* (*Les coupures et les fermetures des bibliothèques de santé canadiennes : ce que fait l'ABSC/CHLA, et ce que vous pouvez faire aussi*). Il explore les récentes activités de plaidoyer de l'ABSC/CHLA au nom de ses membres et partage des approches de plaidoyer que ceux et celles d'entre nous qui jouent un rôle en information de la santé peuvent également mettre en pratique.

Ce numéro contient trois articles de recherche:

- Alexander, Hall et Chen explorent l'implication des bibliothécaires dans les articles de synthèse des connaissances et sa relation avec le nombre de citations et le facteur d'impact d’une revue.
- McKeown, Hopman et Roy comparent l'enseignement en personne dans une salle de classe à l'enseignement vidéo en ligne pour initier les étudiants de troisième cycle aux techniques de recherche documentaire.
- Visintini et McEwan présentent des stratégies simples d'évaluation de l'espace en fonction de l'expérience utilisateur dans le contexte d'une petite bibliothèque d'hôpital afin de soutenir la modernisation du mobilier et les politiques de la bibliothèque.

Dans la section critiques de livres, Leonard examine *Essential leadership skills for health sciences information professionals* (2023) de Crum et Nunez, tandis que Nault fait un compte rendu de l’ouvrage d’Andrea Jamison *Decentering whiteness in libraries: a framework for inclusive collection management practices* publié en 2024.

Notre numéro s'achève sur une analyse par Hilton de *Critical appraisal checklist for systematic reviews and research syntheses* par le Joanna Briggs Institute, qui examine son utilité dans l'évaluation des méthodologies de recherche des revues systématiques.

J'espère que vous apprécierez ce numéro du JABSC/JCHLA!

**Jessica McEwan**

*Directrice de la rédaction JABSC/JCHLA*

*Courriel:* editor@chla-absc.ca

